# Implementation of maternal guidelines for gravid teenagers with hypertensive disorders in KwaZulu-Natal

**DOI:** 10.4102/curationis.v46i1.2395

**Published:** 2023-01-23

**Authors:** Zwelihle B. Shongwe

**Affiliations:** 1 Department of Health, KwaZulu-Natal College of Nursing, Empangeni, South Africa; 2 Department of Health Studies, College of Human Sciences, University of South Africa, Pretoria, South Africa

**Keywords:** gravid teenagers, hypertension, hypertensive disorders in pregnancy, maternal guidelines, teenager

## Abstract

**Background:**

The worldwide phenomenon of teenage pregnancy among 13–9-year-olds is complicated by obstetric conditions. Among the top three causes of maternal mortality, hypertension is the third in South Africa. Quality maternal care is assured by obstetric practitioners (OPs) implementing guidelines specific for management of hypertension in pregnancy.

**Objective:**

The objective of this study was to investigate implementation of maternal guidelines for hypertension in pregnancy among teenagers.

**Methods:**

As a retrospective quantitative research design was used, 173 maternal records of pregnant teenagers from 13 to 19 years were sampled from six district hospitals and Community Health Centres (CHCs) between 01 January 2017 and 31 December 2019 to undergo systematic random sampling. A pretested structured checklist was used to record data from sampled maternal records. Statistical Package for Social Sciences (SPSS) version 26 was used for data analysis, and results were presented using simple descriptive statistics.

**Results:**

Research results indicated that teenagers who suffered from hypertension intrapartum and postpartum did not receive maternal care according to the guidelines for maternity care in South Africa. Blood pressure was not measured of six (3.47%) intrapartum and five (2.9%) postpartum teenagers. Seventeen (9.8%) hypertensive postpartum teenagers received their antihypertensives.

**Conclusion:**

Public health institutions (PHIs) compromised provision of quality maternal care among teenagers, evidenced by incomplete intrapartum and postpartum assessment, diagnosis and management of hypertensive disorders in pregnancy (HDP).

**Contribution:**

This study contributed to facilitating adherence to guidelines improving healthcare of teenagers in government facilities.

## Introduction

Teenage pregnancy, that of a pregnant woman aged 13–19 years, affects all countries (Sanchez & Favara 2019:12). Starting sexual acts before 20 years increases maternal, foetal and neonatal complications caused by variety of diseases such as hypertensive disorders in pregnancy (HDP), commonly affecting teenagers (Dewau, Mekonnen & Seretew 2021:5). The teenage women’s reproductive system matures at about 12–14 years old, predisposing them to HDP, which remains in the top three causes of maternal death worldwide (Berhe et al. 2020:2; Waugh & Grant 2018:495; World Health Organization [WHO] 2020:1).

Butalia, Audibert and Cote (2018:526) spell out that per 100 000 live births, 17% of maternal deaths occurred in the United States (US), 7% in Canada and 0.38% between 2010 and 2012 occurred in the United Kingdom (UK) because of HDP. South Africa (SA) records 210 maternal deaths from hypertension-related conditions, with 31 maternal deaths from KwaZulu-Natal while six per 100 000 live births occurred in the Western and Northern Cape during the 2017 calendar year. South Africa reports maternal deaths due to the scope of care for a designated public health facility where mortality occurred. Five maternal deaths occurred in community health centres (CHCs), whereas 34 and 65 occurred in district and regional hospitals, respectively (South Africa 2017:66).

To minimise complications associated with HDP among teenagers for obstetric practitioners (OPs) to apply their knowledge and experience when implementing obstetric guidelines for assessment, diagnosis and management (Peres, Mariana & Cairrao 2018:9). Worldwide, obstetric departments implement various guidelines for HDP. The US, UK and Canada implement the American College of Obstetricians and Gynecologists’ (ACOGs) opinions, the National Institute for Health and Care Excellence (NICE) guidelines and Canada’s 2018 Guidelines for the Management of Hypertension in Pregnancy, while South Africa applies the 2016–2018 maternal guidelines on HDP (ACOG Committee opinion 2018:e44, 2019:e174; Butalia et al. 2018:526; NICE 2019:1).

Guidelines of HDP apply to adults and teenagers to guide assessment, diagnosis and management, thus improving quality maternal care during pregnancy, labour and puerperium. Obstetric practitioners need to assess the risks of hypertension when a teenager experiences labour pains and after delivery of the foetus. The parameters to be assessed include measuring blood pressure (BP), assessing and grading oedema, testing urine using dipsticks and so forth (South Africa 2016:30). This study aimed at investigating implementation of maternal guidelines for hypertensive disorders among teenagers, during intrapartum and postpartum periods in KwaZulu-Natal.

## Research methods and design

### Research design

A nonexperimental retrospective quantitative study was conducted in order to determine the quality of the maternal care rendered on the basis of assessment and diagnosis of hypertensive disorders among intrapartum and postpartum teenagers. The documented maternal care in the maternal records was used by the researcher to justify type of care rendered on the basis of the Guidelines for Maternity Care in South Africa (2016). In a retrospective design, the researcher uses past recorded information from maternal records to investigate a current phenomenon and reach a conclusion, allowing generalisation of the results (Patten & Newhart 2018:13). This study used the maternal records of teenagers who suffered from HDP. These maternal records were from the period between January 2017 and December 2019. The researcher retrospectively assessed strategies applied to implement guidelines for HDP among teenagers in the province of KwaZulu-Natal.

### Setting

The researcher conducted this study in a natural setting in KwaZulu-Natal, consisting of a CHC and six district hospitals in a rural district. These public health institutions (PHIs) render various healthcare services, including antepartum, intrapartum and postpartum care. The district consisted of urban, semi-urban and deep rural areas regulated by traditional tribal authorities.

### Study population

The study population were maternal records between 01 January 2017 and 31 December 2019 for teenagers who were living with HDP and treated for HDP during pregnancy, labour and puerperium. Maternal records of teenagers aged 13–19 years indicated those who were living with HDP from January 2017 and December 2019 for their inclusion in this study. To retrieve these records, a list with admission numbers from admission and discharge registers of teenagers diagnosed with HDP was used by the administrative personnel.

### Sampling

The researcher used the sampling frame comprising admission numbers between 01 January 2017 and 31 December 2019 to start a systematic random sampling process (Polit & Beck 2021:269). During this process, the first randomly selected element recorded was followed by selecting every third element until the required sample size was achieved. The calculated sample size, using a margin error of 0.005% and a 95% confidence level, was 159 and increased by 15% for nonresponses to achieve a final sample size of 173. Administration clerks at the research sites assisted with retrieval of 173 maternal records, using a list with admission numbers.

### Data collection

The researcher developed a new structured checklist using the Guidelines for Maternity Care in South Africa (2016) and the latest obstetric literature on important elements for HDP. A structured checklist, arranged in a systematic manner, consisted of demographics, history collected during antenatal care, morbidity during pregnancy, problems manifested during pregnancy, history of labour and postpartum assessment and care.

### Data analysis

The quantitative recorded data were analysed using descriptive statistics. According to Patten and Newhart (2018:203), a researcher considers distribution before summarising descriptive data. Using code numbers such as 1, 2, 3, 4 and 5, the simplified classification of data was entered into an Excel spreadsheet (Microsoft Corporation, Redmond, Washington, United States) for analysis. Cleansing of data for completeness and correctness was performed, and data were analysed using the Statistical Package for the Social Sciences (SPSS) version 26.0 (IBM Corporation, Armonk, New York, United States). Polit and Beck (2021:383) recommend that descriptive data be presented in numeric and graphic form using contingency tables, bar graphs and pie charts. The study results were presented using frequency tables including percentages.

### Ethical considerations

Ethical clearance to conduct this study was obtained from the University of South Africa Health Studies Higher Degrees Ethics Review Committee (ref. no. HSHDC/994/2020). The Department of Health in KwaZulu-Natal, district managers and selected public health institutional managers officially permitted this study. Codes were used instead of real names of institutions and maternal records to maintain confidentiality and anonymity. The signing of a confidentiality agreement form between the statistician and the researcher strengthened confidentiality in this study. The PHIs reserved small spaces dedicated for the researcher to record data on site.

The admission numbers of teenagers extracted from the admission and discharge registers kept in labour wards were used to form a comprehensive list, which was submitted to the administration clerks for retrieval of maternal records. The signing of a borrowing register by the researcher while receiving and returning maternal records to administrative personnel prevented loss of these files after use, and 11–48 maternal records were recorded per day.

### Measures of validity and reliability

#### Validity

Validity means the accuracy of the data collection instrument to measure what was supposed to be measured (Polit & Beck 2021:806). The internal validity of this study was enhanced through a pilot study and consistency during data collection, without altering the structured checklist. External validity was maintained by generalising results only in PHIs which were selected for this study

#### Reliability

Reliability refers to the degree of dependability of a data collection instrument to yield similar results when repeatedly used by other researchers on the same population (Polit & Beck 2021:806). The reliability of this study was enhanced through the services of a statistician on Cronbach’s alpha testing for 48 variables in a structured checklist. The good score was 0.711, assuring the accuracy and consistency of the structured checklist used in this study to measure what was meant to be measured.

## Results

### Biographics of the sample

The implementation of the maternal guidelines for hypertension in pregnancy was assessed from 173 maternal records of teenagers who suffered from hypertensive disorders during intrapartum and postpartum periods. Table 1 presented features on the biographics of the sample. The seven research sites selected were six district hospitals and a CHC. The ages of the teenagers were categorised into four strata, with the first stratum consisting of one (0.58%), the youngest teenager at 13 years; 14 (8.09%) in the age stratum of 14–15 years; 48 (27.75%) in the age stratum of 16–17 years; and 110 (*n* = 63.58%) in the stratum of 18–19 years. In the sample, the majority of teenagers were pregnant for the first time, while 7.5% (*n* = 13) were pregnant for the second time; 96.53% (*n* = 167) had no foetal loss, with few teenagers experiencing miscarriages.

**TABLE 1:** Biographic features of the sample.

Variables	Frequency (*N* = 173)	Percentage
**1. Records sampled per health institution**		
Community health centre - 1	20	11.60
District hospital - 2	13	7.50
District hospital - 3	26	15.00
District hospital - 4	11	6.40
District hospital - 5	30	17.30
District hospital - 6	25	14.50
District hospital - 7	48	27.70
**2. Teenagers’ ages in years**		
13	1	0.58
14–15	14	8.09
16–17	48	27.75
18–19	110	63.58
**3. Gravida status**		
Primigravida	160	92.49
Multigravida (Gravida 2)	13	7.51
**4. Previous foetal loss**		
No foetal loss	167	96.53
Foetal loss through miscarriage	6	3.47

## Research findings

### Intrapartum and postpartum maternal assessment

The severity of HDP depends on intrapartum and postpartum assessment findings from various body systems. In this study, intrapartum and postpartum assessments were focused on cardiovascular and urinary systems. These assessments measured BP, assessment and grading of oedema and urinalysis tests. Table 2 displays various assessments with study results.

**TABLE 2:** Intrapartum and postpartum assessment of hypertension among teenagers.

Assessment	Frequency (*N* = 173)	%
**Intrapartum assessment**		
Measuring blood pressure		
Not measured	6	3.47
70–139/50–105	117	67.63
140–224/70–134	46	26.59
Rechecked	4	2.31
Urinalysis test		
Not performed	52	30.10
No proteinuria	109	63.00
Proteinuria ≥ 1+	12	6.90
Assessment of oedema		
Not assessed	170	98.27
Assessed and graded	3	1.73
**Postpartum assessment**		
Measuring blood pressure
Not measured	5	2.90
90–139/40–96	151	87.30
140–159/80–100	15	8.70
170–174/110–114	2	1.10
Urinalysis test		
Not carried out	172	99.40
No proteinuria	1	0.60
Assessment of oedema		
Not assessed	172	99.40
Assessed and graded	1	0.60

### Intrapartum assessment

#### Measuring blood pressure

The heart pumps blood at a normal pressure, and this pressure can be estimated and called the BP. This study found that the blood pressure was not measured in 6 (3.47%) of the teenagers.

#### Urinalysis test

To detect diseases of the urinary system, the urine needs to be tested using various tests such as urinalysis. The urinalysis test results showed no proteinuria in 63.03% (*n* = 109); 6.94% (*n* = 12) had proteinuria, and others had no urinalysis test performed.

#### Oedema

Swelling in the body manifests a disease that needs specific management. Out of 173 teenagers who experienced labour pains and were admitted in PHIs, only 1.73% (*n* = 3) were assessed for oedema, but it was not assessed in the majority of teenagers.

### Postpartum assessment

#### Measuring blood pressure

In this study, the values of BP were grouped into four strata, with the first stratum consisting of teenagers whose BP was not measured; the second stratum consisted of teenagers who had a normal BP; the third stratum (*n* = 15, 8.7%) had BP of 140–159/80–100 mmHg; and the fourth stratum had BP of 170–174/110–114 mmHg.

#### Urinalysis test

The study found 0.58% (*n* = 1) of teenagers had a urinalysis test after delivery.

#### Assessment of oedema

Out of 173 teenagers who delivered in the PHIs, only 0.6% (*n* = 1) had oedema assessed and graded, and the majority were not assessed.

### The management of hypertension among teenagers during and after labour

Hypertensive disorders in pregnancy require therapeutic treatment intrapartum and postpartum to prevent maternal–foetal–neonatal complications. This study was focused on the management of HDP during labour and after delivery, as the determinant of the mode of delivery and maternal and neonatal condition after delivery, which might affect the duration of hospital stay postpartum. Table 3 shows the results of intrapartum and postpartum maternal and neonatal care and complications.

**TABLE 3:** Intrapartum and postpartum care and complications.

Intrapartum	Frequency (*N* = 173)	%
**Gestational age at onset of labour**		
BBA	3	1.73
Unsure of dates	4	2.31
Miscarriage	3	1.73
≥ 28–36 weeks	22	12.72
37–42 weeks	141	81.50
**Received antihypertensives**		
Received intrapartum	22	12.72
Not received intrapartum	151	87.28
Received postpartum	17	9.80
Not received postpartum	156	90.20
**Method of delivery**		
NVD with episiotomy cut	105	60.69
NVD without episiotomy cut	25	14.45
Caesarean section	43	24.86
**Neonate condition at birth**		
Alive	168	97.11
Demised after birth	5	2.89
**Apgar scoring at birth**		
3/10 – 0/10	5	2.89
6/10 – 10/10	2	1.16
7/10 – 10/10	13	7.51
8/10 – 10/10	153	88.44
**Maternal postpartum complications**		
No complications	152	87.86
New onset of HDP	2	1.15
Eclampsia	1	0.58
Retained placenta	1	0.58
PPH	2	1.15
Vaginal tears	14	8.10
Anaemia	1	0.58
**Neonatal complications**		
No complications	172	99.42
Neonatal jaundice	1	0.58
**Maternal length of stay in facility post-delivery**		
< 6 hours	1	0.58
≥ 6 h ≤ 2 days	115	66.50
3–5 days	56	32.40
Not recorded	1	0.58

HDP, hypertensive disorders in pregnancy; NVD, normal vaginal delivery; BBA, birth before arrival; PPH, postpartum haemorrhage.

#### The gestational age at the onset of labour

Normal labour starts spontaneously from 37 to 42 completed weeks’ gestation including limited interventions when a teenager delivers vaginally (Sellers 2018:369). The study found 141 (81.50%) pregnant teenagers who had normal labour while others experienced miscarriages and delivered prematurely.

#### Treatment of eclampsia during labour and after delivery

Antihypertensives prescribed intrapartum and postpartum normalise BP. This study found 22 (12.7%) teenagers received antihypertensives during labour, and others did not receive any treatment related to hypertension in pregnancy. Again, 17 (9.8%) received antihypertensives after delivery while the majority did not receive such treatment.

#### Method of delivery

Delivery of teenagers commonly needs to be assisted with other means to be effective; otherwise, complications may occur. The majority of teenagers delivered vaginally with episiotomy cuts, but 25 (14.45%) gave birth without episiotomy cuts, and others were delivered by caesarean section.

#### Condition of the baby after birth

Foetuses need to adjust in the extra-uterine environment, and effective or ineffective adjustment is mostly indicted by a scoring instrument. This study found five (2.89%) babies who demised after birth, while others survived. The findings also reveal that 153 (88.44%) had a normal Apgar score of 8/10 in 1 min and 10/10 in 10 min, and other babies had a varying degree of asphyxia neonatorum.

#### Maternal complications

The HDP induced various complications, some preventable while others were manageable intrapartum and postpartum. The study found two (1.16%) teenagers who had developed hypertension during the fourth stage, two (1.16%) who developed postpartum haemorrhage (PPH) and less than 1% who had eclampsia, anaemia and retained placenta.

#### Neonatal complications

Other foetuses fail to adjust to the extra-uterine environment and develop complications. In this study, only one (0.58%) neonate had developed jaundice, which was treated with phototherapy, and others were without complications.

### Maternal length of stay in facility after delivering

Teenagers need to stay in the facility after delivery to monitor their condition for the development of life-threatening complications. The results revealed that 115 (66.5%) teenagers were discharged after 6 h until the second day postpartum, 56 (32.4%) teenagers stayed for 3–5 days, and the others were discharged after less than 6 h.

## Discussion

### Intrapartum and postpartum assessment

The study focused on teenagers aged 13–19 years who became pregnant with their bodies still in the process of growth. To be pregnant at an early age increases the risks of developing conditions complicating pregnancy among teenagers. Such conditions include anaemia, HDP and infections (particularly sexually transmitted), leading to foetal loss. Risk of HDP increases three times higher among teenagers aged less than 15 years. According to Abebe et al. (2020:4), teenagers are 2.29 times more likely to develop HDP.

Obstetric practitioners assess intrapartum and postpartum teenagers for cardinal signs of HDP. These are hypertension, proteinuria and oedema. The Government of the Republic of South Africa standardised guidelines for routine assessment of these cardinal signs during labour and after delivery (Sellers 2018:291; South Africa 2016:30). These assessments are cost-effective and simple to perform as they require less expensive equipment, while others do not need equipment anymore. The study found six (3.47%) and five (2.9%) teenagers who had no BP measured, while 170 (98.27%) and 172 (99.4%) teenagers were not assessed for oedema during labour and after delivery. Onyeajam et al. (2018:4) and Van Pelt et al. (2020:5), in their studies, and the KZN Department of Health Annual Report 2020–2021 (2022:51) report and emphasise that insufficient medical resources for screening, a lack of knowledge, negative staff attitude towards HDP and shortage of OPs compromise quality maternal care in Tanzania and KwaZulu-Natal.

According to South Africa (2016:73), facility managers and OPs must keep available resources to assess HDP for specific management. This allows specific management to be rendered. The main focus is to save the teenager, and thereafter, the foetus. Shongwe and Shakwane (2022:11) concluded that incomplete assessments of HDP, shortage of material resources and partial adherence to principles of record-keeping compromise quality in maternal care among adolescents aged 13–19 years. Certain factors such as gestational age determine the type of care that saves the mother and foetus, although delivery of the products of conception decreases the severity of hypertension. Obstetric practitioners need to consider complications associated with prematurity while deciding a teenager should deliver vaginally. The study found only 22 (12.72%) teenagers who delivered vaginally before term. This was supported by Kassa et al. (2019:8), who reported 51 (14.1%) teenagers who delivered preterm babies vaginally.

To help teenagers deliver, OPs cut episiotomies, assisting a premature foetal head in distending the vulva while preventing vaginal tearing and excessive moulding from the resistance of pelvic floor muscles against stretching. Cutting episiotomies can be contraindicated for other teenagers, particularly those who test human immunodeficiency virus (HIV)-positive during pregnancy or labour, minimising mother-to-child HIV transmission. Findings from this study revealed that the majority (60.69%, *n* = 105) of teenagers were given episiotomies. These findings are higher than those recorded by Abebe et al. (2020:4), who reported 30.7% (*n* = 95) teenagers who underwent episiotomy during delivery, meaning teenagers should be assisted with episiotomies to hasten the second stage of labour. Without episiotomies, teenagers have an increased likelihood of tearing in the birth canal, predisposing them to complications such as PPH and infection after birth. Abebe et al. (2020:4) found 7.4% (*n* = 23) of teenagers, compared to 5.2% (*n* = 16) of adults, who sustained vaginal tears during birth. This study reported 8.1% (*n* = 14) of teenagers who sustained vaginal tears.

Countries implement various guidelines recommending periods of stay in the health facility after delivery. During this period, OPs conduct assessments for early recognition of life-threatening conditions. In this study, 66.5% (*n* = 115) were admitted from 6 h to 2 days. South Africa (2016:75) supports these results as it is recommended that teenagers who suffered from HDP must be observed in the public health facility for at least 24 h postpartum. Managing HDP during labour determines Apgar scoring of the foetus after birth. The score reflects the type of maternal care rendered and possibilities for foetal survival postdelivery. In this study, 12.72% (*n* = 22) teenagers were suffering from hypertension during labour and received hypertension medication, compared with 11.3% (*n* = 35) teenagers who were found in the study conducted by Abebe et al. (2020:4). Delivering the foetus and placenta lowers the severity of hypertension, thus decreasing incidence of this condition postpartum (ed. Nolte 2018:293). This study supports conclusion made by Nolte (ed. 2018:293), who reported 17 (9.8%) postpartum from 46 (26.59%) intrapartum teenagers who suffered from HDP. Prescribing antihypertensive drugs reduces foetal and neonatal complications induced by maternal hypertension during and after labour. Common neonatal complications include jaundice, anaemia and so on, which mainly affect babies born prematurely (WHO 2020:3). The results of this study revealed that 2.89% (*n* = 5) of babies manifested with severe asphyxia, indicated by a 3/10 – 0/10 Apgar score at 1 and 5 min after birth. These results were similar with those of Kassa et al. (2019:8), which recorded 1.3% (*n* = 5) of babies with Apgar scores less than 6 in 5 min. Asphyxia neonatorum increases the mortality rate (Sellers 2018:380).

### Strengths and limitations

A rural district in KwaZulu-Natal province interested the researcher to focus the study on teenagers who presented themselves in PHIs for intrapartum and postpartum care. To implement principles of retrospective design, data were recorded in a structured checklist from maternal records of teenagers. Data were recorded in the absence of OPs, as the researcher is a midwifery specialist with understanding in obstetric-related data. The researcher presented results generalised to intrapartum and postpartum teenagers suffering from HDP and seven research sites included in this study. In future, further studies need to focus on OPs’ knowledge and experience in implementation of guidelines for hypertension in pregnancy among teenagers and explore perceptions and experiences of teenagers living with HDP.

### Recommendations

The effective implementation of guidelines for hypertension in pregnancy depends on the academic knowledge and experience of OPs. The OPs need to undergo continuous professional development through formal education, workshops, and so forth on hypertension in pregnancy. The researcher recommends future research to focus on developing a midwifery clinical competency model as an effective strategy in the management of hypertensive disorders intrapartum and postpartum among teenagers.

## Conclusion

The determinants of quality assurance include OPs and adequate material resources. Incomplete assessments from a lack of obstetric-required material cause OPs to fail in providing quality maternal care. This results in nonadherence to the 2016 Guidelines for Maternity Care in South Africa. Obstetric practitioners need to be excellent in maternal care through complete and relevant assessments, diagnoses and management of hypertension in pregnancy to reduce morbidity and mortality among future heroines of the South African nation.
